# Ocular infection with *Gliocladium* species—report of a case

**DOI:** 10.1186/s12348-017-0128-1

**Published:** 2017-03-14

**Authors:** Ramesh Venkatesh, Prachi Gurav, Manisha Agarwal, Neelam Sapra, Prachi Abhishek Dave

**Affiliations:** 1 Retina & Vitreous Department, Dr Shroff Charity Eye Hospital, 5027, Kedarnath Road, Daryaganj, New Delhi, 110002 India; 2 Ocular Microbiology Department, Dr Shroff Charity Eye Hospital, 5027, Kedarnath Road, Daryaganj, New Delhi, 110002 India

**Keywords:** Buckle infection, *Gliocladium* species, Fungus

## Abstract

**Purpose:**

The purpose of this study is to report a case of ocular infection with *Gliocladium* species due to an exposed scleral buckle.

**Design:**

Interventional case report was used as the study design.

**Methods:**

A 60-year-old diabetic male patient presented with persistent pain, redness, and discharge in his left eye since 2 months. He had been treated previously with both topical and systemic steroids for a diagnosis of autoimmune scleritis. He had undergone scleral buckling surgery with cryotherapy for an inferior rhegmatogenous retinal detachment in the past. His best-corrected visual acuity was 6/6, N6 and 6/6, N6 in the right and left eyes, respectively. Retraction of the left lower lid revealed an exposed scleral buckle with an overlying necrotic conjunctiva. Scleral buckle removal was done. Microbiological examination showed *Gliocladium* species growing on blood agar and Sabouraud dextrose agar. Treatment was started with topical antifungal medication and oral antibiotics.

**Results:**

Following treatment, signs of infection showed resolution. Patient underwent retinal reattachment surgery with favorable anatomic and visual outcome.

**Conclusion:**

Ocular infection with *Gliocladium* species has not been previously reported. Poor response to steroids and uncontrolled diabetes should make the clinician aware of a possible fungal infection. Removal of the scleral buckle, identification of the causative organism, and use of appropriate antibiotics are important for the accurate management of the case.

## Findings

### Introduction

In the current era, scleral buckling with explants still remains an important and effective technique to attach the retina. Extrusion and infection of the scleral buckle (SB) are the two most common indications for SB removal [1]. Many organisms, generally bacteria, have been implicated as a cause of SB infection. Fungus as a cause of SB infection is rare. We report a case of ocular infection in an old diabetic man caused by a rare fungus called *Gliocladium* species due to an exposed SB. This happens to be the first reported case of ocular infection due to *Gliocladium* species in literature to the best of our knowledge.

## Case description

**Fig. 1:**
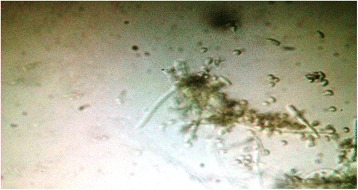
Microscopic examination of *Gliocladium* species. Microscopic examination shows the hyaline hyphae, conidiophores, and conidia borne from hyaline phialides suggestive of *Gliocladium* species

A 60-year-old male was referred to the retina clinic of a tertiary eye care hospital with complaints of persistent pain, watering, and redness in the left eye for the past 2 months. He was treated elsewhere with topical and oral corticosteroids for a diagnosis of possible autoimmune scleritis. He was a known case of insulin-dependent diabetes mellitus with uncontrolled blood sugar at the time of presentation to the retina clinic. He underwent scleral buckling surgery with cryotherapy in that eye for an inferior rhegmatogenous retinal detachment elsewhere 2 years ago. Operative notes of the left eye suggested a no. 276 SB explant placed in the inferior quadrant along with a 360° encircling band. Examination revealed a best-corrected visual acuity (BCVA) of 6/6, N6 and 6/6, N6 in the right (RE) and left eyes (LE), respectively. Anterior segment examination of the RE was normal. Nuclear cataract was noted in the RE. Fundus examination of the RE was normal. Lid edema and conjunctival congestion was noted in the LE. Careful retraction of the left lower lid showed a necrotic conjunctiva in the infero-temporal quadrant with an underlying exposed SB element. Patient was pseudophakic in the LE. Fundus examination showed a shallow recurrent inferior retinal detachment in the LE. However, the retina at the macula was attached. A diagnosis of exposed and infected SB was made, and a decision to explant the SB element along with scleral patch graft was taken. Cefotaxime injection 1 g i.v. twice a day and gentamicin injection 60 mg i.v. thrice a day were given a day before the surgery. Intraoperatively, the SB along with the encircling band was removed under local anesthesia with maximal sedation. The buckle material was a solid silicone rubber which was removed after cutting the anchoring sutures of the SB to the sclera. No evidence of scleral thinning was noted, and the globe was well formed. Scleral patch graft was not required. Postoperatively, the patient was started on empirical antibiotics; tablet ciprofloxacin 750 mg twice a day, topical moxifloxacin eye drops 10 times a day, and topical lubricants four times a day. The scleral buckle grew *Gliocladium* species on both blood agar and Sabouraud dextrose agar (SDA). Microscopic examination showed hyaline hyphae and conidiophores suggestive of *Gliocladium* species (Fig. 1). Based on the culture growth, topical antibiotics were changed to topical voriconazole eye drops 10 times a day, and topical lubricants four times a day were continued. Post-operative examination of the left eye in 1 month showed a quiet eye with no conjunctival congestion. Fundus evaluation of the left eye showed an inferior macula-on retinal detachment for which he underwent three port pars plana vitrectomy with endolaser and gas endotamponade. On his last follow-up visit, his BCVA in the left eye remained stable at 6/6, N6 with a completely attached retina.

## Discussion

SB infection and extrusion remain fairly uncommon complications following scleral buckling surgery. Their estimated incidence varies from 0.2 to 5.6% [2–6]. Coagulase-positive and coagulase-negative *Staphylococci* are implicated as the most common organisms causing SB infection (70 to 90% of cases) [6]. However, rarer cases with infections due to atypical mycobacterium, corynebacteria, and fungi have been reported [7]. In a series of 132 cases of SB infections studied by Chhablani et al. [2], only 15 cases were caused by fungi. The most common fungus isolated in their series was the *Aspergillus* species. Our patient was an elderly diabetic male, misdiagnosed as autoimmune scleritis, and treated extensively with topical and oral corticosteroids. Careful examination revealed an exposed SB element with an overlying necrotic conjunctiva. Culture positivity was noted in 5 days. The SB grew *Gliocladium* species on culture media. *Gliocladium* is a mitosporic, filamentous fungus. Commonly occurring species include *Gliocladium penicilloides*, *Gliocladium virens*, and *Gliocladium roseum*. Most species of *Gliocladium* grow rapidly in culture producing spreading colonies with a cotton-like texture, covering a petri dish in 1 week. The colonies are initially white and cream-like but may become reddish or green as they age and sporulate. Microscopically, *Gliocladium* species produces hyphae, conidiophores, and conidia borne from hyaline phialides. The conidiophores are erect, dense, and have a brush-like structure which produces tapering, slimy phialides [8]. *Gliocladium* species is a saprophytic fungus. The necrotic conjunctiva and scleral tissue provides an excellent environment for the organism to grow. In our case, following the growth of this rare fungus, we rightly modified our treatment by adding topical voriconazole to the regime.

## Conclusion

To the best of our knowledge, this is the first case report of ocular infection with *Gliocladium* species. Poor response to steroids and uncontrolled diabetes should make the clinician aware of a possible fungal infection. Removal of the SB, identification of the causative organism, and use of appropriate antibiotics are vital for the accurate management of the case.

## Abbreviations

BCVA: Best-corrected visual acuity
i.v: Intravenous
LE: Left eye
RE: Right eye
SB: Scleral buckle
SDA: Sabouraud dextrose agar
